# Graphical interface for goodness-of-fit evaluation and clustering of probability distributions in environmental datasets

**DOI:** 10.1016/j.mex.2025.103586

**Published:** 2025-08-27

**Authors:** Felix Reba, Toha Saifudin, Rimuljo Hendradi

**Affiliations:** a Doctoral Program of Mathematics and Natural Sciences, Faculty of Sciences and Technology, Universitas Airlangga, Surabaya, Indonesia; b Mathematics Department, Faculty of Sciences and Technology, Universitas Airlangga, Surabaya, Indonesia; c Information Systems Study Program, Faculty of Science and Technology, Universitas Airlangga, Surabaya, Indonesia

**Keywords:** Chlorophyll, Probability distributions, Model evaluation, K-means, Mixture model, Model accuracy, Marine environment

## Abstract

Goodness-of-Fit (GoF) tests are applied to assess the suitability of probability distributions for environmental data. However, classical methods such as Kolmogorov–Smirnov (KS) and Anderson–Darling (AD) often yield inconsistent outcomes in heterogeneous datasets. Previous studies employed clustering or mixture modeling separately, lacking integration with automated estimation and adaptive weighting. This study introduces a unified framework combining GoF evaluation, K-Means++ clustering, and a KS-weighted mixture model to enhance distribution selection. Seventeen univariate probability distributions were tested on chlorophyll concentration data from the Black Sea, with adequacy assessed via KS and AD tests and five information criteria. The framework was implemented via a MATLAB GUI to automate clustering, estimation, model selection, and evaluation steps. Tested across multiple sample sizes and extended to variables, the GUI demonstrated adaptability and robustness. Model performance showed that the KS-weighted mixture model provided stable fits for complex datasets, improving interpretability and reducing reliance on single-distribution assumptions. *This study supports SDG 14 by enhancing tools for monitoring marine ecosystem health through robust modeling of chlorophyll concentration, a key proxy for marine environmental status.*

Integrates GoF testing, clustering, and mixture modeling

Implements a reproducible workflow via MATLAB GUI

Enhances robustness and positions mixture modeling within environmental data analysis

## Graphical abstract

The graphical abstract illustrates the end-to-end workflow implemented in the MATLAB-based GUI, including input selection, GoF evaluation, cluster validation (Silhouette and DBI), model performance assessment using RMSE and KL Divergence, and final visualization of mixture distribution results and clustering metrics.

**Figure:**
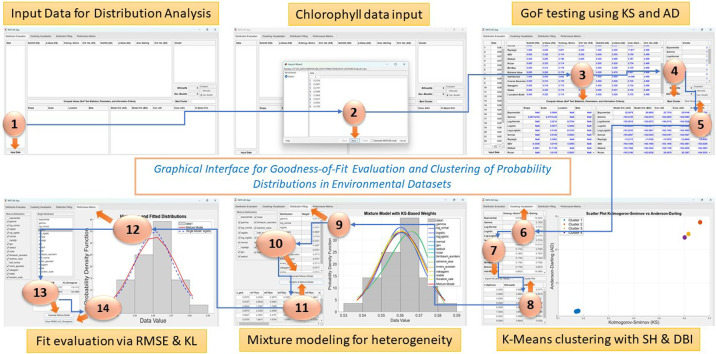

## Specifications table

**Table:** 

**Subject area**	Mathematics and Statistics
**More specific subject area**	Goodness-of-Fit Evaluation, K-Means Clustering, Mixture Modeling, MATLAB GUI for Environmental Data.
**Name of your method**	GoF–Clustering–Mixture Model Framework via MATLAB GUI
**Name and reference of original method**	The method builds upon prior works integrating goodness-of-fit testing, clustering, and mixture e modeling. It is adapted from: Van & Pham-Gia (2010), Clustering probability distributions. da Silva et al. (2023), Mixture models of probability distributions applied to rainfall in the state of Pernambuco, Brazil. Pata et al. (2024), Data-driven determination of zooplankton bioregions and robustness analysis. These studies provided the statistical foundation and data-driven techniques extended in the present GUI-assisted framework.
**Resource availability**	Raw chlorophyll dataset available from the Copernicus Marine Service: https://data.marine.copernicus.eu. MATLAB GUI software and processed data available upon reasonable request from the corresponding author.

## Background

Univariate probability distributions play a central role in diverse scientific fields, including wind analysis, extreme climate prediction, environmental risk assessment, and health surveillance, by enabling clearer interpretation of complex univariate data [[1], [2], [3], [4]]. In marine studies, they are particularly valuable for modelling chlorophyll concentration, which serves as a proxy for plankton biomass and ecosystem dynamics [5]. However, real-world environmental datasets often display heterogeneity, skewness, and multimodality, making it difficult for any single theoretical distribution to accurately capture their characteristics [4].

This study evaluates the suitability of 17 continuous univariate distributions for modelling chlorophyll concentration data, an indicator of plankton activity in marine systems [6,7]. The candidate distributions range from the Exponential and Gamma to the Stable and t Location–Scale families, as listed in Table 1. Previous studies have frequently applied distributions such as Gamma, Lognormal, Log-logistic, and Weibull in environmental contexts [4,8]. Goodness-of-fit (GoF) tests, including the Kolmogorov–Smirnov (KS) and Anderson–Darling (AD) tests, are commonly employed to evaluate model adequacy [[9], [10], [11]]. However, these tests can produce inconsistent results, particularly with small or skewed samples, introducing uncertainty into model selection [12].

**Table 1:** Probability distribution models and their parameters.

Distribution	Distribution Model	Parameters
Exponential	$f\left(x\right)=\frac{1}{\theta }\exp\left(\frac{-x}{\theta }\right)$	θ=scale
Gamma	$f\left(x\right)=\frac{x^{a-1}}{b^{a}\Gamma \left(a\right)}\exp\left(-\frac{x}{b}\right)\;$	α=shape b=scale
Log Normal	$f\left(x\right)=\frac{1}{x\sigma \sqrt{2\pi }}\exp\left\{\frac{-{\left(\log x-\mu \right)}^{2}}{2\sigma^{2}}\right\}$	μ=location σ=scale
Logistics	$f\left(x\right)=\frac{\exp(\frac{x-\mu }{\sigma })}{\sigma {\left(1+\exp\left(\frac{x-\mu }{\sigma }\right)\right)}^{2}}$	μ=location σ=scale
Log Logistics	$f\left(x\right)=\frac{1}{\sigma x}\frac{\exp(\frac{\ln\;x\;-\mu }{\sigma })}{{\left(1+\exp(\frac{\ln\;x\;-\mu }{\sigma })\right)}^{2}}$	μ=location σ=scale
Normal	$f\left(x\right)=\frac{\exp(-\frac{1}{2}{\left(\frac{x-\mu }{\sigma }\right)}^{2})}{\sigma \sqrt{2\pi }}\;$	μ=location σ=scale
Rayleigh	$f\left(x\right)=\frac{x}{b^{2}}\exp\left(-\frac{1}{2}\frac{x^{2}}{b^{2}}\right)$	b=scale
GEV	$f\left(x\right)=\frac{1}{\sigma }{\left[1+\xi (\frac{x-\mu }{\sigma })\right]}^{-\frac{1}{\xi }-1}\exp\left\{-{\left[1+\xi (\frac{x-\mu }{\sigma })\right]}^{-\frac{1}{\xi }}\right\}$	μ=location σ=scale ξ=shape
Weibull	$f\left(x\right)=\frac{b}{a}{\left(\frac{x}{a}\right)}^{b-1}\exp\left(-{\left(\frac{x}{a}\right)}^{b}\right)$	α=scale b=shape
Rician	$f\left(x\right)=I_{o}\left(\frac{\mathrm{xs}}{\sigma^{2}}\right)\frac{x}{\sigma^{2}}\exp-\left(\frac{x^{2}+s^{2}}{2\sigma^{2}}\right)$	s=location σ=scale
Birnbaum Saundars	$f\left(x\right)=\frac{1}{\sqrt{2\pi }}\exp-\frac{{\left(\sqrt{\frac{x}{\beta }}-\sqrt{\frac{\beta }{x}}\right)}^{2}}{2\gamma^{2}}\frac{\sqrt{\frac{x}{\beta }}+\sqrt{\frac{\beta }{x}}}{2\gamma x}$	β=scale γ=shape
Extreme Value	$f\left(x\right)=\sigma^{-1}\exp\left(\frac{x-\mu }{\sigma }\right)\exp\left(-\exp(\frac{x-\mu }{\sigma })\right)$	μ=location σ=scale
Half Normal	$f\left(x\right)=\sqrt{\frac{2}{\pi }}\frac{1}{\sigma }\exp-\frac{1}{2}{\left(\frac{x-\mu }{\sigma }\right)}^{2}$	μ=location σ=scale
Inverse Gaussian	$f\left(x\right)=\sqrt{\frac{\lambda }{2\pi x^{3}}}\exp\left\{-\frac{\lambda }{2\mu^{2}x}{\left(x-\mu \right)}^{2}\right\}$	λ=shape μ=scale
Nakagami	$f\left(x\right)=2{\left(\frac{\mu }{\omega }\right)}^{\mu }\frac{1}{\Gamma (\mu )}x^{\left(2\mu -1\right)}\exp\left(\frac{-\mu }{\omega }x^{2}\right)$	μ=shape ω=scale
Stable	$E\left(e^{\mathrm{itX}}\right)=\left\{\begin{array}{c} \exp(-\gamma^{\alpha }{\left|t\right|}^{\alpha }\left[1+i\beta \mathrm{sign}\left(t\right)\tan\frac{\pi \alpha }{2}\left({\left(\gamma |t|\right)}^{1-\alpha }\right)-1\right])+i\delta t\\ \exp(-\gamma |t|[1+i\beta \mathrm{sign}\left(t\right)\frac{2}{\pi }\ln\left(\gamma |t|\right)]+i\delta t) \end{array}\right\}$	α=shape β=shape γ=scale δ=location
t Location Scale	$f\left(x\right)=\frac{\Gamma (\frac{v+1}{2})}{\sigma \sqrt{v\pi }\Gamma \left(\frac{v}{2}\right)}{\left[\frac{v+{\left(\frac{x-\mu }{\sigma }\right)}^{2}}{v}\right]}^{-(\frac{v+1}{2})}$	μ=location σ=scale υ=shape

Recent work has attempted to address these challenges, though important gaps remain. For instance, Pata et al. [13] employed clustering techniques to delineate zooplankton bioregions based on species abundance but did not apply probability distribution modelling to environmental indicators such as chlorophyll. Similarly, da Silva et al. [14] fitted mixture models of probability distributions to rainfall data in Pernambuco, Brazil. Although this study addressed heterogeneity through multiple candidate distributions and the KS test, it did not incorporate information-theoretic scoring criteria such as the Akaike Information Criterion (AIC) and Bayesian Information Criterion (BIC), nor did it employ clustering to assess similarities among fitted models. Moreover, neither study proposed a reproducible computational framework for environmental data modelling.

**Fig. 1:**
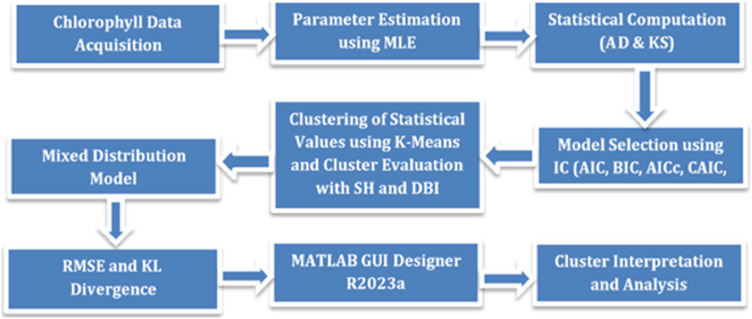
illustrates the structured workflow, beginning with chlorophyll data input and continuing through parameter estimation, Goodness-of-fit (GoF) testing, model selection, clustering, mixture modeling, model fitting, and final interpretation using an integrated GUI-based system.

The present study addresses these methodological and practical gaps by introducing a multilevel framework that includes: (1) GoF-based evaluation of 17 univariate distributions using KS and AD tests; (2) integration of AIC and BIC to enhance model robustness; (3) application of k-means clustering to group distributions by statistical similarity; and (4) mixture modelling to account for distributional heterogeneity. To support these tasks, a MATLAB-based graphical user interface (GUI) is developed, enabling parameter estimation, GoF testing, model comparison, clustering, and visualization within a reproducible and user-friendly environment (Fig. 1). The framework is applied to chlorophyll data to enable more consistent and flexible environmental modelling, with root mean square error (RMSE) and Kullback–Leibler divergence (KL divergence) employed to quantitatively assess model fit quality.

In addition, this study contributes to Sustainable Development Goal (SDG) 14: Life Below Water, particularly Target 14.1, by enhancing tools for monitoring marine ecosystem health. By improving statistical modeling of chlorophyll concentration, a key proxy for plankton biomass and nutrient dynamics, the proposed framework aids in assessing eutrophication and pollution trends in marine environments.

## Method details

### Probability and probability distributions in environmental data

Chlorophyll concentration data in marine ecosystems frequently exhibit positive skewness, high variability, and long-tailed behavior, which limit the applicability of discrete probability models such as the Poisson or Binomial distributions [5,15]. Continuous probability distributions—such as Gamma, Lognormal, and Weibull—are generally more suitable because of their flexibility in representing asymmetric patterns and heavy-tailed characteristics [16,17]. Similar considerations apply to other environmental variables, including rainfall intensity, river discharge, and pollutant concentrations, which typically display non-negative values, high skewness, and occasional extreme events.

Seventeen continuous probability distributions were selected based on their documented relevance in the environmental modeling literature and their suitability for skewed, non-normal data. This set includes widely used models such as Gamma, Lognormal, Weibull, Exponential, Gumbel, and the Stable distribution, among others (see Table 1 for the full list). Specific distributions were chosen for their ability to represent particular data characteristics—for example, Gamma and Lognormal for highly skewed positive datasets; Gumbel and the Generalized Extreme Value (GEV) distribution for modeling extremes; and the Normal or Student’s t distribution for approximately symmetric data [16,17].

All models were fitted to the chlorophyll dataset using maximum likelihood estimation (MLE), implemented through custom MATLAB scripts. Parameter estimation was conducted using MATLAB’s fitdist function [18], which generates initial parameter values from the dataset’s descriptive statistics (e.g., mean, standard deviation, and skewness). These initial estimates were then optimized iteratively to maximize the likelihood. To ensure numerical stability and physically meaningful results, lower bounds were imposed on scale parameters to maintain positive values, while shape parameters were constrained within ranges informed by preliminary data exploration (e.g., 0.1–10) and guidance from the literature.

For each fitted model, Goodness-of-fit (GoF) evaluation was performed using the Kolmogorov–Smirnov (KS) and Anderson–Darling (AD) tests [[9], [10], [11]]. In addition, information-theoretic criteria such as the Akaike Information Criterion (AIC) and Bayesian Information Criterion (BIC) were computed to support model comparison [19,20]. These metrics were used to assess fit quality and as input for k-means clustering, which grouped structurally similar distributions (as elaborated in the clustering subsection). Among the models tested, the Gamma and Lognormal distributions performed well in capturing chlorophyll variability, consistent with previous studies linking these models to plankton dynamics and marine biogeochemical processes [[20], [21], [22]].

A MATLAB-based graphical user interface (GUI) was developed using App Designer to automate the entire modeling process, providing a reproducible and user-friendly environment for fitting, ranking, and clustering models.

To select the most appropriate probability distribution model for chlorophyll data, this study applies multiple Information Criteria (IC) to balance model fit and complexity. Specifically, we used the Akaike Information Criterion (AIC) [23], Bayesian Information Criterion (BIC) [24], Corrected AIC (AICc) [25], Consistent AIC (CAIC) [26], and the Hannan–Quinn Criterion (HQC) [27]. These metrics support objective model comparison and form the basis for subsequent clustering analysis.

### Clustering of probability distributions based on GoF statistics using K-Means

This study employed two Goodness-of-fit (GoF) tests—the Kolmogorov–Smirnov (KS) and Anderson–Darling (AD) tests—to evaluate the statistical compatibility between observed chlorophyll data and theoretical distribution models [14]. The KS statistic measures the maximum absolute deviation between the empirical and theoretical cumulative distribution functions:

(1) $$D_{calculated}=sup_{x}\left|F\left(x\right)-H\left(x\right)\right|$$

The AD statistic, which places greater emphasis on differences in the distribution tails, is given by:

(2) $$A_{n}^{2}=-n-\frac{1}{n}\;\sum_{i=1}^{n}\left(2i-1\right)\left[\log F\left(x_{i}\right)+\log(1-F\left(x_{n+1-i}\right)\right]$$

Using both KS and AD values as clustering features provides complementary sensitivity: KS captures the largest absolute deviations across the distribution, whereas AD is more sensitive to tail behavior.

Clustering was performed using the k-means algorithm, with Euclidean distance applied for computational efficiency:

(3) $$d_{ik}=\sum_{j=1}^{m}{\left(x_{kj}-c_{ij}\right)}^{2}$$

To enhance clustering stability and avoid local minima, the k-means++ initialization method was employed [18]. This approach improves centroid selection and reduces the required iterations compared with standard random initialization. Each clustering run was repeated with 9 replicates to balance stability with computational efficiency.

Clustering performance was evaluated for k values ranging from 2 to 10, reflecting a plausible range for grouping the tested distributions while avoiding overfitting. For each k, the configuration with the highest Silhouette coefficient and lowest Davies–Bouldin Index (DBI) was selected [28,29].

The average Silhouette coefficient is defined as:

(4) $$\bar{S}=\frac{1}{n}\sum_{i=1}^{n}\left(\frac{b\left(i\right)-a\left(i\right)}{\max\left(a\left(i\right),b\left(i\right)\right)}\right)$$

where a(i) is the average intra-cluster distance and b(i) is the distance to the nearest neighboring cluster. A higher Silhouette score indicates stronger cohesion.

The DBI evaluates inter-cluster compactness and separation:

(5) $$IDB=\frac{1}{k}\sum_{i=1}^{k}R_{i}$$

where $R_{i}$​ measures the average similarity between cluster i and its most similar cluster. Lower DBI values indicate superior clustering performance.

The evaluation (Table 5) showed that k=4 provided consistent optimal values for both metrics across all sample sizes and was therefore selected as the final configuration.

Integrating GoF test statistics with k-means++ clustering enables grouping of probability models based on statistical behavior, thereby enhancing both interpretability and reproducibility in model selection.

### Mixture model

To improve clustering reliability when individual distributions yield similar Goodness-of-fit (GoF) values, this study applies a mixture modeling approach. This method overcomes the limitations of selecting a single best-fitting distribution per cluster, which is often inadequate for environmental datasets characterized by high variability.

Previous studies, such as da Silva et al. [14], have shown that no single distribution consistently captures rainfall characteristics across different periods and locations in Pernambuco, Brazil. Building on this insight, we implemented a mixture model that combines several continuous probability distributions into a single integrated framework:

(6) $$f_{mixture}\left(x\right)=\sum_{i=1}^{N}w_{i}f_{i}\left(x\right)$$

In this formulation, $f_{i}\left(x\right)\;$is the probability density function (PDF) of the i-th distribution, $w_{i}$ represents its weight (subject to the constraint $\sum w_{i}=1$), and N is the number of selected distributions. The weights were normalized to ensure that the resulting mixture remains a valid probability distribution, with each $w_{i}$ reflecting the relative contribution of its component.

In this study, the Kolmogorov–Smirnov (KS) statistic was chosen as the primary criterion for evaluating mixture models, owing to its nonparametric nature and its ability to measure the maximum absolute difference between empirical and theoretical cumulative distribution functions. This property makes KS particularly suitable for comparing complex distributional shapes—such as those produced by mixtures—without assuming a specific underlying distributional form. Unlike information-theoretic measures (e.g., AIC, BIC) that penalize model complexity, the KS statistic directly assesses fit quality across the entire data range, effectively detecting discrepancies in both central and tail regions.

The mixture model was constructed using weights derived from KS-based GoF statistics, ensuring that the contribution of each accepted distribution is proportionally adjusted according to its individual fit quality. Distributions that provide stronger statistical support for the data exert greater influence on the final model, thereby improving representational accuracy and reducing the risk of overfitting commonly associated with selecting a single best-fitting distribution. This approach simultaneously enhances flexibility in capturing spatial and temporal patterns in the data, strengthens robustness, and enables more accurate characterization of environmental variability, while supporting reliable statistical inference in distribution-based analyses.

### Evaluation metrics for model comparison

To quantitatively compare the performance of the fitted single-distribution and mixture models, two statistical metrics were used: the Root Mean Square Error (RMSE) and the Kullback–Leibler (KL) divergence. The RMSE measures the average magnitude of the deviation between observed and estimated values [30] and is defined as:

(7) $$RMSE=\sqrt{\frac{1}{n}}\sum_{i=1}^{n}{\left(y_{i}-{\hat{y}}_{i}\right)}^{2}$$

where $y_{i}$​ and ${\hat{y}}_{i}$​ denote the observed and estimated values, respectively, and n is the total number of observations.

The KL divergence measures the information loss between the observed probability distribution P and the model distribution Q:

(8) $$D_{KL}\left(P|Q\right)=\sum_{i}P\left(i\right)log\left(\frac{P\left(i\right)}{Q\left(i\right)}\right)$$

where P(i) and Q(i) are the probabilities of the observed and model distributions at point i, respectively.

These metrics were used to assess the relative fit quality across different sample sizes and distribution models, offering complementary perspectives: RMSE emphasizes accuracy in value estimation, whereas KL divergence focuses on similarity in distributional shape.

### MATLAB app designer integration in the analytical workflow

This study employs the MATLAB App Designer to streamline and automate each stage of the analytical workflow. The graphical user interface (GUI), developed as an application, enables integrated control of data handling, parameter estimation, statistical evaluation, model selection, clustering, mixture modeling, and interpretation—within a single interactive platform [18]. The structured workflow consists of the following stages:

• Chlorophyll data acquisition.
• Parameter estimation using the Maximum Likelihood Estimation (MLE) method.
• Goodness-of-fit (GoF) testing via Kolmogorov–Smirnov (KS) and Anderson–Darling (AD) statistics.
• Model selection based on information criteria (AIC, BIC, AICc, CAIC, HQC).
• Clustering of statistical values using the k-means algorithm, with cluster evaluation based on Silhouette coefficient and Davies–Bouldin Index (DBI) scores.
• Application of mixture distribution models to accommodate heterogeneity in the data.
• Evaluation of model fit quality using RMSE and KL divergence metrics.
• Visualization and interpretation of final analytical outputs.

To enhance reproducibility and usability, the GUI incorporates predefined default parameters for each analytical stage, including:

• Initial parameter values for distribution fitting (automatically generated from descriptive statistics).
• Recommended bounds for scale and shape parameters to ensure physical validity (e.g., positive scale values, realistic shape ranges).
• Optimal clustering settings such as iteration limits and k-value ranges.

Input fields are validated to ensure that entries remain within acceptable limits, thereby reducing computational errors. In addition, the GUI allows users to modify initial estimates, adjust clustering parameters, and change the weighting scheme in the mixture model. This flexibility enables both less experienced and advanced users to tailor the analysis without compromising reliability.

To further improve model reliability and minimize manual error, the GUI integrates clustering visualization through scatterplots of KS and AD statistics, thereby identifying distributional clusters based on statistical similarity. Silhouette and DBI values are also presented in tabular format to support clearer evaluation of cluster cohesion and separation.

As illustrated in Fig. 1, the workflow begins with data acquisition and progresses through sequential steps: parameter estimation via MLE, GoF evaluation, model selection using multiple information criteria, clustering with k-means++ (evaluated using the Silhouette coefficient and Davies–Bouldin Index [DBI] scores), application of mixture models to account for heterogeneity, and final model fit assessment using RMSE and KL divergence metrics. This process is executed within the MATLAB GUI, ensuring consistent, reproducible, and automated application across different datasets while reducing manual errors.

## Method validation

### Research data

The chlorophyll dataset used in this study was obtained from the Copernicus Marine Service [31], focusing on a fixed point in the Black Sea at coordinates 31.14015°E, 41.69411°N. Sampling was conducted at multiple depths and pressure levels to capture vertical variability in chlorophyll distribution throughout the water column.

Prior to analysis, the data were filtered using a probability-domain approach to detect and remove outliers, thereby ensuring statistical validity. The cleaned dataset was then evaluated against 17 candidate continuous probability distributions: Exponential, Gamma, Lognormal, Logistic, Log-logistic, Normal, Rayleigh, Generalized Extreme Value (GEV), Weibull, Rician, Birnbaum–Saunders, Extreme Value, Half Normal, Inverse Gaussian, Nakagami, Stable, and t Location–Scale. Distributions whose domains did not cover the observed range were excluded to avoid parameter estimation bias.

To assess the robustness of the model selection framework under varying data conditions, four sample sizes were tested: n=17,18,29, and56. These sizes were chosen based on observed environmental variability related to depth and pressure and align with standard recommendations for statistical analysis in environmental modeling. According to Monte Carlo simulations reported by Yazici and Yolacan [32], sample sizes in this range are sufficient to reduce inference error and ensure reliable distributional assessments.

A clustering-based validation strategy was also employed, in which distributions were grouped based on similarity in Goodness-of-fit (GoF) test statistics, namely the Kolmogorov–Smirnov (KS) and Anderson–Darling (AD) tests. This approach reduces model selection subjectivity and supports the identification of statistically consistent and interpretable clusters across different sample sizes. Validation outcomes are detailed in the following subsections, including test statistics and clustering performance. To evaluate the generalizability of the developed GUI beyond chlorophyll data, the analysis was extended to include additional environmental variables such as Colored Dissolved Organic Matter (CDOM) and seawater salinity, thereby validating the method’s applicability across diverse oceanographic parameters.

### Test statistic values

The Goodness-of-Fit (GoF) test results presented in Table 2, along with the AIC-based evaluation in Table 3, demonstrate substantial performance variation among 17 candidate probability distributions when applied to empirical chlorophyll datasets across four different sample sizes (*n* = 17, 18, 29, and 56). The distributions evaluated include Exponential, Gamma, Log Normal, Logistic, Log Logistic, Normal, Rayleigh, GEV, Weibull, Rician, Birnbaum–Saunders, Extreme Value, Half Normal, Inverse Gaussian, Nakagami, Stable**,** and t Location Scale.

**Table 2:** Results of KS and AD tests for various distributions.

Distribution	Kolmogorov-Smirnov (KS)				Anderson-Darling (AD)			
	*n* = 17	*n* = 18	*n* = 29	*n* = 56	*n* = 17	*n* = 18	*n* = 29	*n* = 56
Exponential	$H_{1}$	$H_{1}$	$H_{1}$	$H_{1}$	$H_{1}$	$H_{1}$	$H_{1}$	$H_{1}$
Gamma	$H_{0}$	$H_{0}$	$H_{0}$	$H_{0}$	$H_{0}$	$H_{0}$	$H_{0}$	$H_{0}$
Log Normal	$H_{0}$	$H_{0}$	$H_{0}$	$H_{0}$	$H_{0}$	$H_{0}$	$H_{0}$	$H_{0}$
Logistic	$H_{0}$	$H_{0}$	$H_{0}$	$H_{0}$	$H_{0}$	$H_{0}$	$H_{0}$	$H_{0}$
Log Logistic	$H_{0}$	$H_{0}$	$H_{0}$	$H_{0}$	$H_{0}$	$H_{0}$	$H_{0}$	$H_{0}$
Normal	$H_{0}$	$H_{0}$	$H_{0}$	$H_{0}$	$H_{0}$	$H_{0}$	$H_{0}$	$H_{0}$
Rayleigh	$H_{1}$	$H_{1}$	$H_{1}$	$H_{1}$	$H_{1}$	$H_{1}$	$H_{1}$	$H_{1}$
GEV	$H_{0}$	$H_{0}$	$H_{0}$	$H_{0}$	$H_{0}$	$H_{0}$	$H_{0}$	$H_{0}$
Weibull	$H_{0}$	$H_{0}$	$H_{0}$	$H_{0}$	$H_{0}$	$H_{0}$	$H_{0}$	$H_{0}$
Rician	$H_{0}$	$H_{0}$	$H_{0}$	$H_{0}$	$H_{0}$	$H_{0}$	$H_{0}$	$H_{0}$
Birnbaum Saundars	$H_{0}$	$H_{0}$	$H_{0}$	$H_{0}$	$H_{0}$	$H_{0}$	$H_{0}$	$H_{0}$
Extreme Value	$H_{0}$	$H_{0}$	$H_{0}$	$H_{0}$	$H_{0}$	$H_{0}$	$H_{0}$	$H_{0}$
Half Normal	$H_{1}$	$H_{1}$	$H_{1}$	$H_{1}$	$H_{1}$	$H_{1}$	$H_{1}$	$H_{1}$
Inverse Gaussian	$H_{0}$	$H_{0}$	$H_{0}$	$H_{0}$	$H_{0}$	$H_{0}$	$H_{0}$	$H_{0}$
Nakagami	$H_{0}$	$H_{0}$	$H_{0}$	$H_{0}$	$H_{0}$	$H_{0}$	$H_{0}$	$H_{0}$
Stable	$H_{0}$	$H_{0}$	$H_{0}$	$H_{0}$	$H_{0}$	$H_{0}$	$H_{0}$	$H_{0}$
t Location Scale	$H_{0}$	$H_{0}$	$H_{0}$	$H_{0}$	$H_{0}$	$H_{0}$	$H_{0}$	$H_{0}$

**Table 3:** Results of AIC values across four sample sizes (*n* = 17*,*18*,*29*,*56) for various distributions. AIC was selected as the best-performing information criterion among AIC, BIC, AICc, CAIC, and HQC.

Distribution	AIC			
	*n* = 17	*n* = 18	*n* = 29	*n* = 56
Exponential	−10.1842	−9.6936	−27.7631	−40.6920
Gamma	−73.3305	−76.3531	−99.3453	−263.5631
Log Normal	−73.4378	−76.2718	−99.5756	−264.3675
Logistic	−70.9629	−74.6505	−95.7440	−259.3462
Log Logistic	−71.4284	−74.6096	−96.6878	−261.1285
Normal	−72.9520	−76.3289	−98.2471	−261.6158
Rayleigh	−33.2669	−34.1321	−64.9200	−116.9839
GEV	−72.6776	−75.4170	−97.8768	−266.9593
Weibull	−71.7444	−75.9502	−96.9381	−251.0279
Rician	−72.9848	−76.3583	−98.2986	−261.6339
Birnbaum Saundars	−73.4895	−76.3189	−99.7033	−264.4006
Extreme Value	−70.9928	−75.3483	−93.4036	−245.4716
Half Normal	−17.3454	−17.3943	−40.6552	−68.9529
Inverse Gaussian	−73.4901	−76.3185	−99.7052	−264.4035
Nakagami	−73.1834	−76.3845	−98.9844	−262.6632
Stable	−68.9825	−72.3577	−94.2647	−259.8689
t Location Scale	−70.9826	−74.3578	−96.2647	−259.6249

Most distributions exhibited consistent acceptance of the null hypothesis ($H_{0}$) under both the Kolmogorov–Smirnov (KS) and Anderson–Darling (AD) tests. A total of 14 distributions—namely Gamma, Log Normal, Logistic, Log Logistic, Normal, GEV, Weibull, Rician, Birnbaum–Saunders, Extreme Value, Inverse Gaussian, Nakagami, Stable, and t Location Scale—were statistically accepted across all sample sizes, indicating strong structural compatibility with the chlorophyll data. In contrast, the Exponential, Rayleigh, and Half Normal distributions consistently failed the GoF tests due to extremely low p-values and high test statistics. These rejections were further supported by performance assessments based on Information Criteria (IC) metrics.

As shown in Table 2, GoF conclusions were derived by comparing p-values and test statistics from both the KS and AD tests. These values were calculated and visualized using the custom MATLAB GUI interface, as illustrated in Fig. 3, which provides tabular outputs and statistical visualization for each sample size. This feature enhances clarity and transparency in the GoF evaluation process.

Further analysis was conducted using the information-theoretic criteria (IC) results presented in Table 3. Among the five IC metrics applied (AIC, BIC, AICc, CAIC, HQC), AIC was identified as the most effective discriminative criterion. The same 14 Goodness-of-fit (GoF)–accepted distributions also produced the lowest AIC values across all sample sizes, confirming their superior performance in terms of fit quality and parameter efficiency. In contrast, the Exponential, Rayleigh, and Half Normal distributions not only failed the GoF tests but also yielded the highest IC values, reinforcing their inadequacy as candidate models.

Overall, these findings highlight the limitations of single-distribution models in capturing the complexity and variability of environmental datasets, while emphasizing the need for more adaptive approaches such as the mixture model, which is discussed further in the section *Results of the Mixed Distribution Model*.

### Clustering method and evaluation

This study implements a k-means clustering framework to assess similarity among 17 candidate probability distributions. As shown in Table 4, the clustering process was conducted for each sample size (*n* = 17, 18, 29, and 56) using test statistics from the Goodness-of-fit (GoF) tests—Kolmogorov–Smirnov (KS) and Anderson–Darling (AD). These values were used as clustering features to group distributions with comparable GoF performance.

**Table 4:** K-means evaluation with silhouette and DBI for various distributions.

Distribution	Silhouette	DBI
	*n* = 17*,*18*,*29*,*56	*n* = 17*,*18*,*29*,*56
Exponential	Cluster 1	Cluster 1
Gamma	Cluster 4	Cluster 4
Log Normal	Cluster 4	Cluster 4
Logistics	Cluster 4	Cluster 4
Log Logistics	Cluster 4	Cluster 4
Normal	Cluster 4	Cluster 4
Rayleigh	Cluster 3	Cluster 3
GEV	Cluster 4	Cluster 4
Weibull	Cluster 4	Cluster 4
Rician	Cluster 4	Cluster 4
Birnbaum Saundars	Cluster 4	Cluster 4
Extreme Value	Cluster 4	Cluster 4
Half Normal	Cluster 2	Cluster 2
Inverse Gaussian	Cluster 4	Cluster 4
Nakagami	Cluster 4	Cluster 4
Stable	Cluster 4	Cluster 4
*t* Location Scale	Cluster 4	Cluster 4

The k-means algorithm was executed iteratively until a convergence threshold was reached, ensuring stable cluster assignments across iterations. This procedure ensures that the final cluster structure accurately reflects statistical relationships among distributions based on KS and AD results.

Once convergence was achieved, the clustering results were validated using two objective evaluation metrics: the Silhouette coefficient and the Davies–Bouldin Index (DBI). These metrics assess clustering quality in terms of compactness and separation, supporting selection of the optimal number of clusters (k). As shown in Table 5, a configuration of four clusters consistently yielded the highest Silhouette coefficient values (approaching 1.0000) and the lowest DBI values (as low as 0.0024) across all sample sizes, confirming a robust clustering structure.

**Table 5:** Clustering evaluation based on silhouette and DBI.

Number of Clusters	Silhouette				DBI			
	*n* = 17	*n* = 18	*n* = 29	*n* = 56	*n* = 17	*n* = 18	*n* = 29	*n* = 56
2	0.9861	0.9889	0.9536	0.9902	0.1437	0.1236	0.2301	0.1214
3	0.9777	0.9802	0.9994	0.9727	0.1557	0.1459	0.0265	0.1676
**4**	**0.9992**	**1.0000**	**0.9999**	**0.9994**	**0.0129**	**0.0024**	**0.0040**	**0.0106**
5	0.9331	0.8581	0.8835	0.9616	0.0660	0.1419	0.1576	0.1241

Table 4 presents the distribution-to-cluster assignments derived from these evaluations. A total of 14 distributions—Gamma, Lognormal, Logistic, Log-logistic, Normal, Generalized Extreme Value (GEV), Weibull, Rician, Birnbaum–Saunders, Extreme Value, Inverse Gaussian, Nakagami, Stable, and t Location–Scale—were consistently grouped into Cluster 4. These distributions exhibited low and stable KS and AD values across all sample sizes (*n* = 17, 18, 29, and 56), indicating strong statistical compatibility with the empirical chlorophyll data.

In contrast, the remaining three distributions—Exponential, Half Normal, and Rayleigh—were assigned to separate clusters (Clusters 1, 2, and 3, respectively), reflecting fundamental differences in model fit and statistical behavior. These distributions exhibited higher test statistics and low p-values in the GoF evaluation, in agreement with earlier findings shown in Table 2.

The resulting cluster configuration and quality indicators are also visualized via scatter plots of KS vs. AD values and summaries of Silhouette and DBI scores (see Fig. 4). These visualizations provide intuitive insight into the distribution landscape and reinforce the objectivity of the clustering outcomes. This clustering approach enhances model generalizability and ensures consistent grouping across varied sample conditions, thereby supporting more reliable model selection.

## Results of the mixed distribution model

As a continuation of the previous statistical analysis, a mixture distribution model was implemented to provide a more flexible and representative characterization of the chlorophyll dataset. This approach offers an alternative to single probability distributions, which often struggle to capture the structural complexity and heterogeneity inherent in empirical environmental data.

**Fig. 2:**
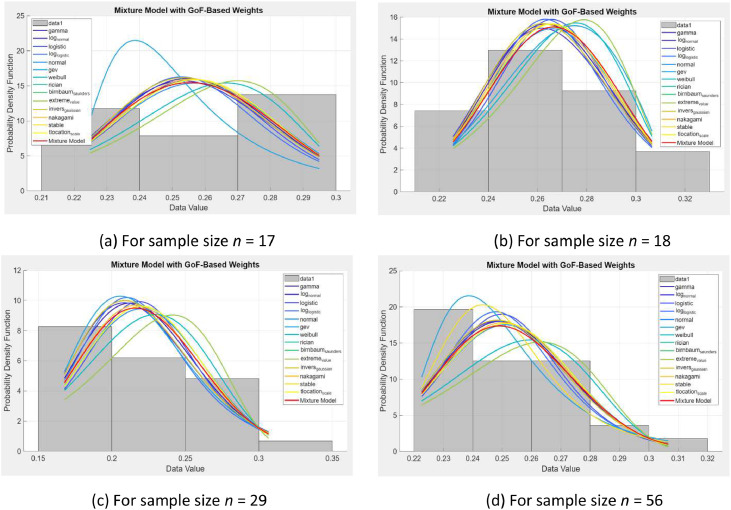
Mixture model and individual distribution plots for four chlorophyll sample sizes (*n* = 17*,*18*,*29, and 56).

Fig. 2 presents overlays of the empirical data histograms with probability density functions (PDFs) from 14 individual distributions that passed the Goodness-of-Fit (GoF) evaluation using both Kolmogorov–Smirnov (KS) and Anderson–Darling (AD) tests. Of the 17 candidate distributions initially assessed, only 14 are visualized. The remaining three—Exponential, Rayleigh, and Half Normal—were excluded due to consistently low p-values and high test statistics, leading to the rejection of the null hypothesis (H₀). The mixture model developed in this study was constructed solely from the accepted distributions, as these distributions demonstrated statistical compatibility with the observed chlorophyll data.

To construct the mixture model, the contribution of each accepted distribution was weighted based on its KS-based GoF performance. This weighting scheme ensures that distributions offering a better statistical fit exert greater influence on the final mixture distribution. Table 6 summarizes the assigned weights for each distribution across the four sample sizes evaluated (*n* = 17, 18, 29, and 56). These weight values were derived directly from the GoF statistics and reflect the relative compatibility of each distribution with the empirical chlorophyll data.

**Table 6:** KS-based weights of candidate distributions used in the mixture model.

Distribution	Weight			
	*n* = 17	*n* = 18	*n* = 29	*n* = 56
Gamma	0.0709	0.0703	0.0705	0.0723
Log Normal	0.0738	0.0747	0.0736	0.0735
Logistic	0.0773	0.0721	0.0747	0.0787
Log Logistic	0.0789	0.0727	0.0741	0.0774
Normal	0.0716	0.0740	0.0715	0.0742
Gev	0.0794	0.0727	0.0725	0.0794
Weibull	0.0649	0.0753	0.0753	0.0574
Rician	0.0692	0.0697	0.0695	0.0729
Birnbaum Saunders	0.0716	0.0703	0.0715	0.0723
Extreme Value	0.0619	0.0686	0.0663	0.0517
Invers Gaussian	0.0716	0.0703	0.0715	0.0723
Nakagami	0.0702	0.0697	0.0700	0.0723
Stable	0.0695	0.0697	0.0695	0.0723
T location Scale	0.0692	0.0697	0.0695	0.0729

These weights reflect the proportional contribution of each accepted distribution in shaping the final mixture distribution for each sample size. The resulting mixture curves, derived from the GoF-based weighting approach, are further illustrated in Fig. 2. This figure presents a visual comparison between the fitted mixture model and the individual component distributions across the four chlorophyll sample sizes, providing insights into how well the composite model captures the empirical data behavior.

**Fig. 3:**
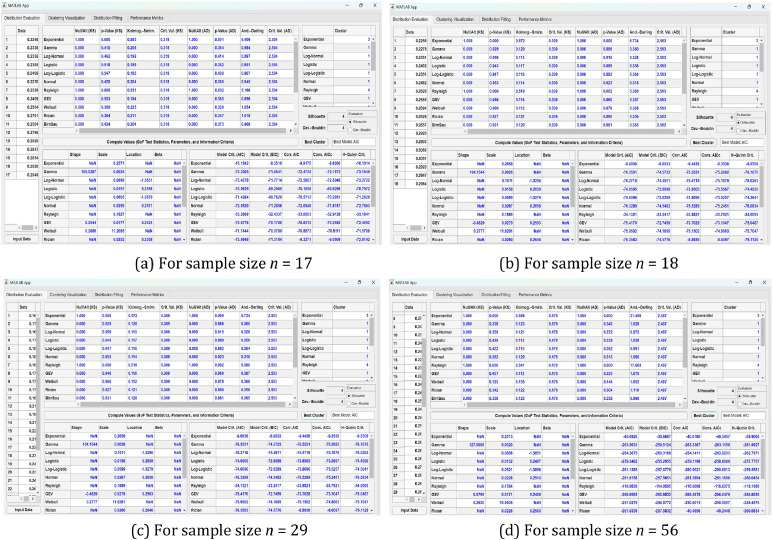
(a–d) MATLAB GUI Designer interface displaying the evaluation of distribution fits based on goodness-of-fit statistics, parameter estimation, and information criteria, along with clustering results and best model selection across different sample sizes (*n* = 17, 18, 29, and 56).

As shown in Fig. 2, Fig. 3, the mixture model curves (highlighted in red) closely align with the empirical data across all four sample sizes (*n* = 17, 18, 29, and 56). Several individual distributions, such as Inverse Gaussian, Nakagami, and Log Normal, demonstrate similar behavior to the mixture model—particularly near the data's central tendency. However, noticeable deviations remain, especially in the tails and peaks, reinforcing the limitations of using single distributions when analyzing environmentally variable data.

Importantly, the mixture model was constructed using weights derived from the KS-based GoF statistics. This weighting approach proportionally adjusts the contribution of each accepted distribution to the final mixture model based on their individual GoF performance. By doing so, distributions that provide a better statistical fit to the data have a greater influence on the resulting model. This enhances the accuracy of the distributional representation and mitigates the risk of overfitting commonly associated with selecting a single best-fitting distribution.

Notably, this approach aligns conceptually with the work of da Silva et al. [14], who applied mixture distribution models to monthly rainfall data in Pernambuco, Brazil. While both studies emphasize the importance of addressing heterogeneity through composite distributions, the present work expands this framework by integrating visual overlay validation, GoF test statistics (KS and AD), and comparative mixture modeling across multiple sample sizes. Moreover, the use of chlorophyll as an environmental indicator introduces a marine-focused dimension that differs from rainfall-based hydrological modeling. Consequently, these findings reinforce the research direction toward adopting mixture-based approaches as a more robust alternative to classical single-distribution methods in environmental data modeling.

Although the mixture model is designed to offer a more flexible representation of heterogeneous data, the evaluation results revealed that some single distributions—particularly those ranked highest based on information criteria such as AIC or BIC (e.g., top 1–2)—yielded lower Root Mean Square Error (RMSE) and Kullback–Leibler (KL) Divergence values than the mixture model. This outcome is expected, as those single models demonstrate strong compatibility with the observed data under specific conditions. In contrast, for lower-ranked single distributions (e.g., ranked 3 or lower), the mixture model consistently produced lower RMSE and KL Divergence values, indicating superior generalization and flexibility across variable data structures.

Furthermore, it was also observed that RMSE and KL Divergence values occasionally diverged in direction, where one metric favored the single model and the other favored the mixture model. This behavior is considered normal, given that RMSE measures pointwise deviations between observed and estimated values, while KL Divergence assesses distributional dissimilarity on a probabilistic scale.

This pattern is illustrated in Fig. 2, which overlays the component distributions within the mixture model along with the empirical data. A specific example highlighting the comparative RMSE and KL Divergence outcomes between the mixture and a selected single distribution is shown in Fig. 6.

### Role of the MATLAB GUI designer

This study utilizes a custom-designed MATLAB GUI to streamline and integrate model evaluation and clustering for empirical environmental data characterized by distributional heterogeneity. As outlined in the structured workflow (Fig. 1), the GUI supports all core analytical stages—chlorophyll data acquisition, parameter estimation via Maximum Likelihood Estimation (MLE), Goodness-of-Fit (GoF) testing (Kolmogorov–Smirnov and Anderson–Darling), model selection using Information Criteria (AIC, BIC, AICc, CAIC, HQC), clustering using k-means, and mixture model implementation.

**Fig. 4:**
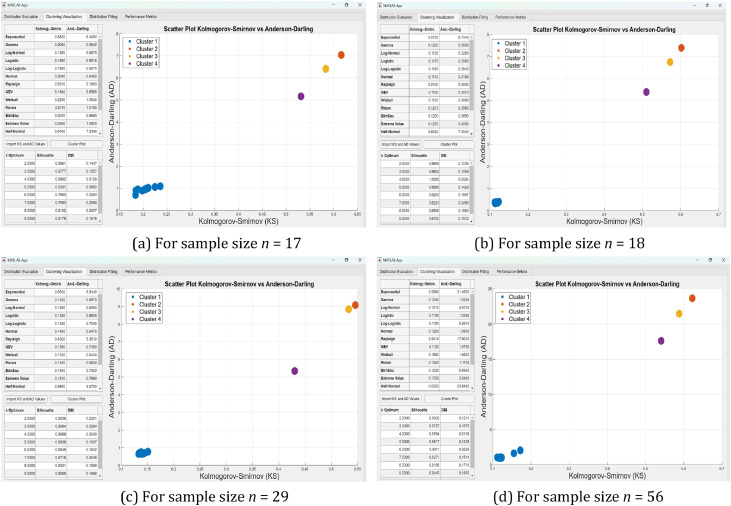
(a–d) Scatter plots of Kolmogorov–Smirnov (KS) versus Anderson–Darling (AD) values with corresponding optimal cluster configurations (k optimum) for distribution groups across different sample sizes (*n* = 17, 18, 29, and 56), as visualized using the MATLAB GUI Designer.

**Fig. 5:**
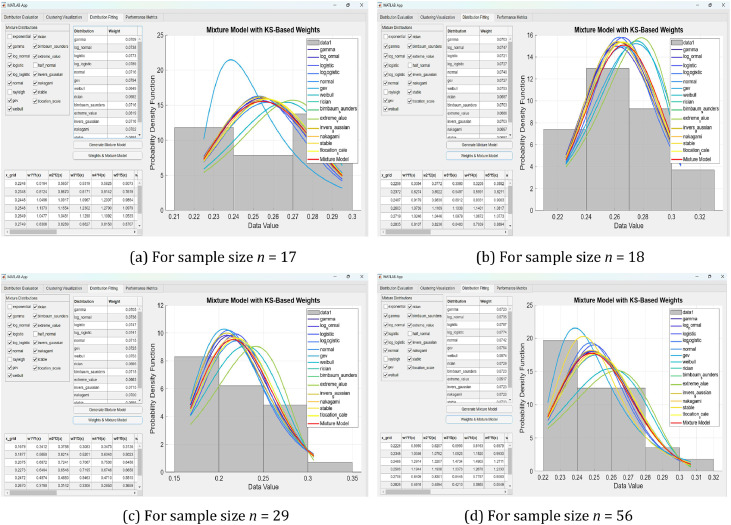
(a–d) Mixture and component distribution visualizations with KS-based weights across sample sizes (*n* = 17, 18, 29, and 56), generated via MATLAB GUI Designer to reflect each distribution’s contribution to overall density.

All functionalities are embedded into an interactive GUI created with MATLAB App Designer, as illustrated in Figs. 3, 4, and 5. The GUI consists of two principal components. The left panel provides interactive access to statistical outputs (e.g., p-values, test statistics, IC values), cluster assignments, and metrics such as Silhouette and Davies–Bouldin Index (DBI). Meanwhile, the right panel visualizes clustering results using scatter plots of KS vs. AD values. This enables clear empirical comparisons across different sample sizes (n=17,18,29, and 56), facilitating more consistent and transparent evaluations of distributional similarity (Fig. 4).

**Fig. 6:**
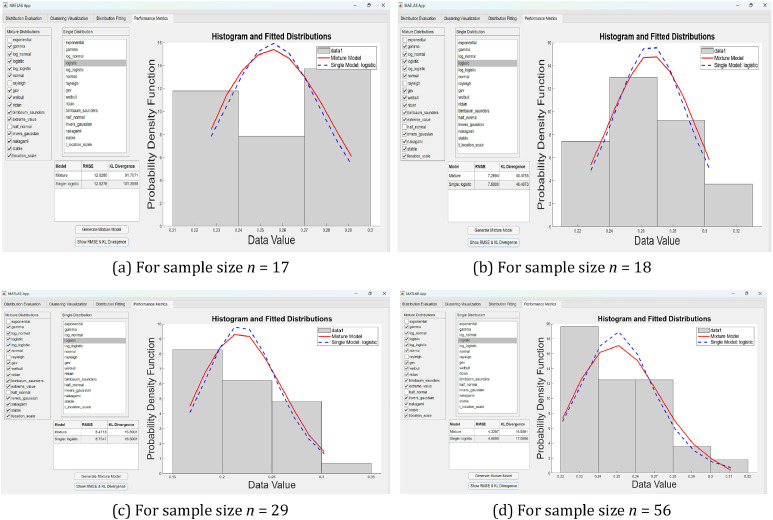
(a–d) Comparison of Mixture Model and Single Logistic Distribution Fitting across Different Sample Sizes (*n* = 17, 18, 29, and 56), Based on RMSE and KL Divergence Metrics, as Visualized Using the MATLAB GUI Designer.

Compared to prior implementations, the GUI offers significant advantages in analytical depth and usability. For instance, past studies such as [13] employed clustering for bioregional definition but lacked visual tools for model validation. Similarly, da Silva et al. [14] applied mixture models to rainfall data using the KS test alone without clustering support. In contrast, the present GUI fully integrates GoF testing (KS and AD), K-Means clustering, IC evaluation, and visual diagnostics. This integration enables simultaneous statistical evaluation and graphical interpretation, increasing transparency and reproducibility in model-based decisions.

Fig. 5 focuses on the final module of the GUI, which presents an overlay of Probability Density Functions (PDFs) from individual distributions and their composite mixture model plotted against empirical histograms. The overlay highlights comparative model accuracy and emphasizes the superior alignment of mixture models, particularly in central and tail regions. This capability is especially useful for environmental datasets, such as chlorophyll concentration, where single distributions often fail to capture spatial and temporal variability. Furthermore, the mixture weights used in these overlays were derived from the GoF statistics (KS-based), reinforcing the statistical foundation of the approach.

Taken together, this GUI-assisted framework supports a robust, visually guided, and data-driven methodology for environmental modeling, facilitating reproducibility and enhancing insight into distributional dynamics.

### Demonstration of GUI on additional environmental datasets

To demonstrate the broader applicability and robustness of the proposed methodology across diverse marine environmental contexts, the analysis was extended beyond chlorophyll concentration to include additional environmental datasets . One such dataset comprises measurements of Colored Dissolved Organic Matter (CDOM) in seawater, expressed in equivalent mass fractions of quinine sulfate dihydrate (unit: 1e–9). This dataset was obtained from the EU Copernicus Marine Service (Product ID: *INSITU_GLO_PHYBGCWAV_DISCRETE_MYNRT_013_030*; Dataset ID: *cmems_obs-ins_glo_phybgcwav_mynrt_na_irr*), and includes in situ observational variables such as geographic coordinates, time, depth, pressure, and recorded CDOM values. In total, 29 observations (*n* = 29) were selected for analysis in this study [31].

The inclusion of this dataset highlights the methodological flexibility of the proposed framework and demonstrates its capability to handle heterogeneous environmental data characterized by varying statistical distributions and structures. This cross-application further validates the generalizability of the developed GUI and supports its use in broader marine data analysis scenarios.

**Fig. 7:**
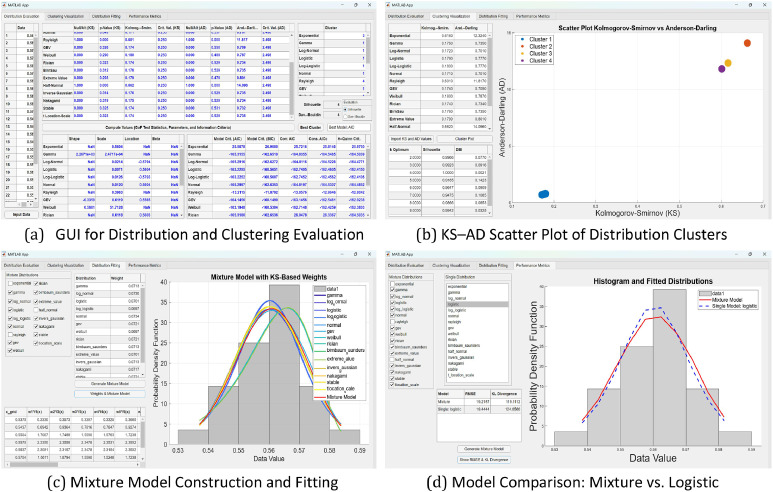
(a–d) MATLAB GUI for mixture model generation and evaluation: distribution fitting, weighting, and model comparison using statistical metrics (RMSE and KL divergence) for the CDOM dataset.

The application of the GUI-based methodology to the CDOM dataset further reinforces the validity of the proposed approach in terms of both robustness and adaptability to varying environmental data. As illustrated in Fig. 7, the distribution evaluation module consistently identified the same 14 candidate distributions that had previously passed the Goodness-of-Fit (GoF) tests for the chlorophyll dataset, while the remaining three distributions—Exponential, Rayleigh, and Half-Normal—were again rejected due to consistently low p-values. This cross-dataset consistency highlights the general reliability of these 14 distributions when applied to univariate environmental data, particularly within the context of distributional behavior analysis.

Furthermore, the weighting scheme used in constructing the mixture model (Fig. 7c) demonstrates the methodological flexibility of the developed GUI. The weights were derived based on the statistical performance of each distribution according to the KS test, allowing those with better fit to contribute more substantially to the overall mixture. The evaluation results reveal that the mixture model derived from the CDOM dataset delivered statistical performance that was comparable to, or better than, the best-fitting single distribution (Fig. 7d), consistent with previous findings from the chlorophyll dataset. This mixture-based approach consistently produced a smoother and more representative distribution curve, particularly in capturing subtle characteristics of the empirical data.

The lower KL divergence and RMSE values further confirm that the mixture model offers a more comprehensive and accurate characterization of the data compared to a single distribution approach. These findings indicate that the proposed methodology is not only valid for a single dataset but can also be effectively transferred to other marine environmental parameters without compromising statistical rigor.

### Ecological and statistical implications

Methodologically, the development of the MATLAB-based Graphical User Interface (GUI) in this study enhances conventional approaches by providing a transparent, practical, and reproducible analytical platform. Unlike previous studies [13] and [14], which did not incorporate clustering techniques or visual diagnostics, the proposed framework offers a more comprehensive means of exploring and validating distribution models. The GUI integrates scatter plot visualisation, statistical summaries, and clustering results to improve model interpretability, particularly in cross-disciplinary applications where users may have varying levels of technical expertise.

Numerous MATLAB-based GUI frameworks have been developed across scientific fields to support domain-specific analysis and data visualisation. For example, [33] introduced a GUI for generating hydrogeochemical diagrams, thereby improving efficiency in geochemical interpretation. In rock mechanics, RSFit3000 [34] enables the estimation of rate–state frictional parameters from experimental data. The MBA-GUI developed by [35] supports chemometric analysis through multi-block data visualisation, regression, classification, and preprocessing tools. In bioacoustics, DetEdit [36] provides a robust platform for annotating and editing long-term acoustic monitoring data. Similarly, CFLab [37] offers a GUI-based tool for analysing sediment grain-size distributions using Weibull functions, thereby advancing sedimentological studies.

Collectively, these tools highlight the adaptability and value of MATLAB-based GUI environments in delivering interactive and domain-specific analytical workflows. They also provide relevant benchmarks for the integrated statistical GUI framework introduced in this study.

### Methodological limitations and adaptation conditions

Although the proposed workflow demonstrated strong performance on the tested dataset, several methodological limitations should be considered when applying it to other contexts. The approach assumes that the input dataset is sufficiently large and representative to enable reliable parameter estimation and model comparison. When sample sizes are small, data are strongly skewed, or distribution tails are underrepresented, the statistical power of Goodness-of-fit (GoF) tests and the stability of parameter estimation may be reduced.

The method also relies on the assumption of independence among observations, which may be violated in datasets exhibiting strong temporal or spatial autocorrelation. In such cases, adjustments such as block bootstrapping, pre-whitening, or alternative GoF measures may be required to maintain validity. For mixture models, the Kolmogorov–Smirnov (KS)-based weighting scheme may require recalibration when data characteristics differ substantially from those examined in this study—for example, in multimodal distributions or when extreme outliers dominate the tails.

To adapt this workflow to a variety of environmental datasets, users should evaluate model assumptions early in the analysis and adjust parameter bounds, clustering ranges, and weighting criteria as necessary. Incorporating additional model selection approaches, such as cross-validation or Bayesian methods, may further improve reliability under complex data conditions. These adaptations ensure that the workflow remains applicable across diverse datasets while maintaining interpretability and reproducibility.

## Conclusions

This study introduces a MATLAB-based graphical user interface (GUI) framework that integrates parameter estimation, goodness-of-fit (GoF) testing, information criteria, K-means clustering, and mixture modeling to enable rigorous and reproducible univariate distribution analysis for environmental data. When applied to chlorophyll concentration datasets across multiple sample sizes (*n* = 17, 18, 29, 56), the framework demonstrated strong capability in addressing data heterogeneity and improving model selection accuracy. Fourteen distributions consistently satisfied GoF criteria, while Exponential, Rayleigh, and Half-Normal were rejected due to poor fit. Clustering results were robust, as indicated by high Silhouette scores and low Davies–Bouldin Index (DBI) values, with an optimal cluster number (k=4) consistently identified. Mixture models weighted by Kolmogorov–Smirnov (KS) scores also outperformed or matched the best-fitting single distributions in terms of RMSE and Kullback–Leibler (KL) divergence, particularly for capturing multimodal behavior and complex distributional features in chlorophyll data.

The generalizability of the framework was further validated using additional datasets, including colored dissolved organic matter (CDOM) and seawater salinity from the EU Copernicus Marine Service. Across these datasets, 14 distributions were consistently retained, reaffirming the reliability of the model selection approach. The GUI’s modular design ensures transparency, reproducibility, and ease of use for researchers in both environmental and marine sciences. Its integrated visual outputs, clustering analytics, and support for multiple sample sizes make it an adaptable tool for analyzing a wide range of univariate environmental datasets. The flexibility of the platform also allows potential future extensions to spatial modeling, time-series analysis, and other oceanographic parameters.

In addition to its methodological contributions, this study supports the realization of Sustainable Development Goal (SDG) 14: Life Below Water, particularly Target 14.1, which aims to reduce marine pollution and eutrophication. By improving statistical modeling of key marine indicators—such as chlorophyll and CDOM—the framework contributes to more accurate monitoring of ocean health and data-driven marine conservation efforts. This positions the proposed GUI not only as a robust analytical tool but also as a practical support mechanism for sustainability-focused research and policy implementation in marine environments.

## Limitations

The method may be less effective for environmental datasets that do not exhibit heterogeneity or multimodal behavior. It assumes the availability of continuous variables with sufficient sample sizes. Application to discrete or sparse datasets may limit performance.

## Ethics statements

This study used publicly available data and did not require ethical approval.

Data availability: The chlorophyll dataset analyzed during the current study is publicly available from the Copernicus Marine Service (https://data.marine.copernicus.eu/). Additional processed data and the MATLAB GUI software developed in this study are available from the corresponding author upon reasonable request.

## Declaration of generative AI and AI-assisted technologies in the writing process

During the preparation of this manuscript, the author(s) used ChatGPT (developed by OpenAI) to assist in improving the clarity, grammar, and academic tone of the English language used in the text. The tool was employed specifically to support language refinement and paraphrasing, while all scientific content, results, interpretations, and conclusions were entirely conceived, written, and validated by the author(s). All AI-assisted output was carefully reviewed and edited, and the author(s) take full responsibility for the final content of the manuscript.

## CRediT authorship contribution statement

**Felix Reba:** Conceptualization, Methodology, Software, Data curation, Writing – original draft, Visualization. **Toha Saifudin:** Supervision, Writing – review & editing, Formal analysis, Project administration. **Rimuljo Hendradi:** Supervision, Validation, Writing – review & editing, Resources.

## Declaration of competing interest

The authors declare that they have no known competing financial interests or personal relationships that could have appeared to influence the work reported in this paper.

## Data Availability

The data supporting the findings of this study are publicly available from the Copernicus Marine Service at .https://data.marine.copernicus.eu
